# Effect of fluorination of naphthalene diimide–benzothiadiazole copolymers on ambipolar behavior in field-effect transistors†

**DOI:** 10.1039/c8ra02915f

**Published:** 2018-05-03

**Authors:** Cunbin An, Hanna Makowska, Benlin Hu, Ruomeng Duan, Wojciech Pisula, Tomasz Marszalek, Martin Baumgarten

**Affiliations:** Max Planck Institute for Polymer Research Ackermannweg 10 55128 Mainz Germany martin.baumgarten@mpip-mainz.mpg.de; Department of Molecular Physics, Faculty of Chemistry, Lodz University of Technology Zeromskiego 116 90-924 Lodz Poland marszalek@mpip-mainz.mpg.de pisula@mpip-mainz.mpg.de

## Abstract

Two naphthalene diimide (NDI)–benzothiadiazole (BT) based conjugated polymers with high molecular weight, P1 and P2, were synthesized by introducing F atoms to modulate the electron-donating ability of the BT moiety. 3-Decyl-pentadecyl branched alkyl side chains were employed and expected to improve the molecular organization and device performance. Both polymers have excellent solubility in common organic solvents. UV-vis-NIR absorption and cyclic voltammetry indicate that the maximum absorption wavelength of P2 is blue-shifted and the HOMO energy level of P2 is decreased in comparison with P1. Two dimensional wide angle X-ray scattering of thin films revealed a similar organization of both polymers. A less balanced transport in field-effect transistors with increased electron mobility of 0.258 cm^2^ V^−1^ s^−1^ and lowered hole transport of 2.4 × 10^−3^ cm^2^ V^−1^ s^−1^ was found for P2. Polymer devices of P1 exhibited a balanced ambipolar transport, with a hole mobility of 0.073 cm^2^ V^−1^ s^−1^ and electron mobility of 0.086 cm^2^ V^−1^ s^−1^.

## Abstract 2

Two naphthalene diimide (NDI)–benzothiadiazole (BT) based conjugated polymers with high molecular weight, P1 and P2, were synthesized and compared for their electron-donating ability by varying F-atoms at the BT moiety.

## Graphical abstract

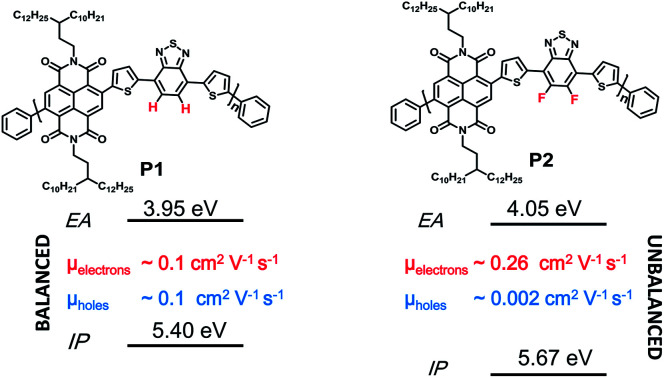

## 1 Introduction

Ambipolar organic field-effect transistors (OFETs) have gained increased attention in the past decade due to their potential in complementary logic circuits.^1–6^ The key design of this kind of polymeric semiconductors is the combination of strong acceptors with suitable donor units in the conjugated backbone, such as naphthalenediimide (NDI),^7–9^ diketopyrrolopyrrole (DPP),^10–13^ thiadiazoloquinoxaline (TQ),^14–16^ and benzobisthiadiazole (BBT).^17,18^ The lowest unoccupied molecular orbital (LUMO) and highest occupied molecular orbital (HOMO) levels are of great importance since they determine the injection and transport of electrons and holes in the active film. High performance ambipolar transistors of donor (D)–acceptor (A) copolymers have been reported with mobilities above 1 cm^2^ V^−1^ s^−1^ for electrons and holes.^19–22^ In order to obtain balanced hole and electron charge carrier mobilities, the energy levels of D–A copolymers need to be finely controlled by adjusting donating and accepting groups in the conjugated backbone. However, it might be challenging for some polymers to attain balanced ambipolar transport by only two structural factors.

To circumvent this problem, one approach is to build polymers with two different acceptor units. Therefore, dual-acceptor architectures have been proposed as D-A1–D-A2 copolymers.^23^ This dual-acceptor architecture provides a higher freedom in tuning the energy levels due to an enhanced degree of structural combinations. Thereby, often stronger acceptors as BBT or TQ possess high electron deficiency providing well balanced ambipolar characteristics *via* combination with weaker acceptors as DPP.^17,24^

The FET performances of conjugated polymers are also dependent on their side chains. The side chain geometries have crucial influence on molecular solubility, packing and thin-film organization, and hence on device performance.^25,26^ For example, the branched alkyl chains can provide better solubility and device performance compared to linear alkyl chains in conjugated polymers.^27^ The branching positions of alkyl chains were also confirmed to reduce the molecular packing distance and enhance device performance.^28^ Therefore, it could be useful strategy to develop well-balanced ambipolar OFETs by using dual-acceptor architecture and tuning branched positions of side chains in conjugated polymers.

**Scheme 1:**
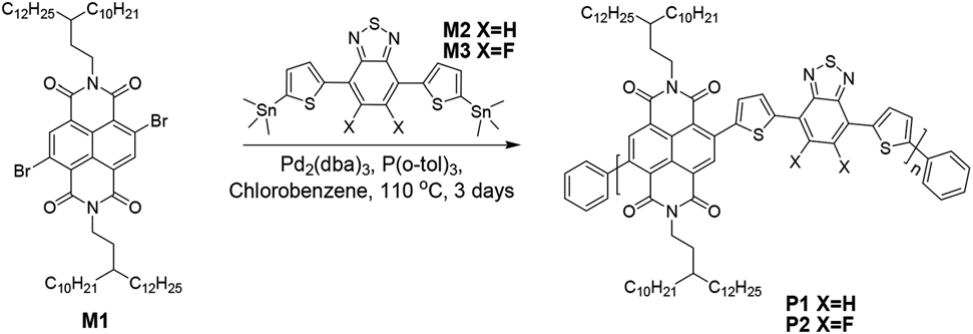
Synthetic route for polymers P1 and P2.

In this work, we describe two dual-acceptor based polymers P1 and P2 (Scheme 1) which are composed of either NDI and benzothiadiazole (BT) or NDI and difluorinated BT (FBT). A pair of 3-decyl-pentadecyl alkyl chains were attached to the NDI unit. They are expected to ensure molecular solubility, strengthen molecular self-organization as well as improve charge carrier transport compared to conventional 2-decyl-tetradecyl substituted NDI.^19,29^ Both polymers reported here, P1 and P2, were compared to each other and to the literature reports regarding their optical and electrochemical properties, self-organization in thin films and charge carrier transport in FETs.

## 2 Results and discussion

### 2.1 Synthesis and characterization

The synthetic route of P1 and P2 is shown in Scheme 1. The synthetic details of monomer M1 is depicted in the ESI.†^30^ Monomers M2 and M3 were prepared according to the literature.^31,32^ Stille coupling reaction was applied to connect M1 with M2 or M3 to obtain the dual-acceptor copolymers. To avoid intermolecular aggregation and obtain reliable molecular weights, high temperature (135 °C) gel permeation chromatography (GPC) was employed using polystyrene as standard and 1,2,4-trichlorobenzene as eluent. The number-average molecular weights (*M*_n_) of 36.8 kg mol^−1^ for P1 and 44.0 kg mol^−1^ for P2 were determined with polydispersity indexes (PDI) of 2.5 and 2.8 for P1 and P2, respectively. Their PDI are much smaller than those of polymers with the same conjugated backbones, but different side chains.^19^ Due to the long branched side chains, both polymers show excellent solubility in chloroform, toluene and chlorobenzene at room temperature (>10 mg mL^−1^). Additionally, an excellent thermal stability was found up to 455 °C with 5% weight loss as shown in Fig. S1† implying that the fluorination does not affect their thermal stability.

### 2.2 Optical and electrochemical properties

**Fig. 1:**
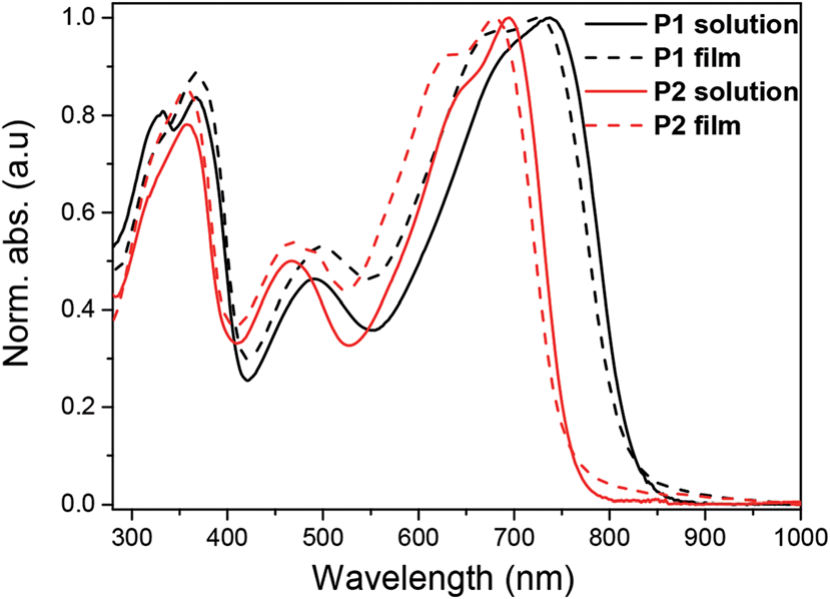
UV-vis absorption spectra of P1 and P2 in chloroform solution (*c* = 10^−6^ M) and thin film.

UV-vis-NIR absorption spectra of the polymers were recorded in diluted chloroform solution (10^−6^ M) as well as in thin film (Fig. 1). In diluted chloroform solution, the absorption spectra profiles of both polymers exhibit three bands, which are similar to other dual-acceptor based polymers.^24^ The region of 300–400 nm and 400–550 nm should origin from π–π* transitions of NDI and BT (or FBT) units, respectively.^8,33,34^ The vis-NIR region (550–850 nm) is attributed to intramolecular charge transfer (ICT) between the NDI and T-BT (or T-FBT) in the polymer backbone. The fluorinated polymer P2 has a significant blue-shift of *λ*_max_ (39 nm) compared to P1, suggesting that the ICT effect of P2 became weaker after the introduction of F atoms into BT unit. This behavior can be well explained by DFT calculations which are discussed below (See 2.3). The films for UV-vis-NIR absorption measurements were prepared by drop-casting onto glass slides from chloroform solution. The polymer thin films displayed very similar absorption bands compared with those in diluted solutions. The optical bandgaps are 1.50 and 1.62 eV, derived from the absorption onset of the solid films for P1 and P2, respectively.

**Fig. 2:**
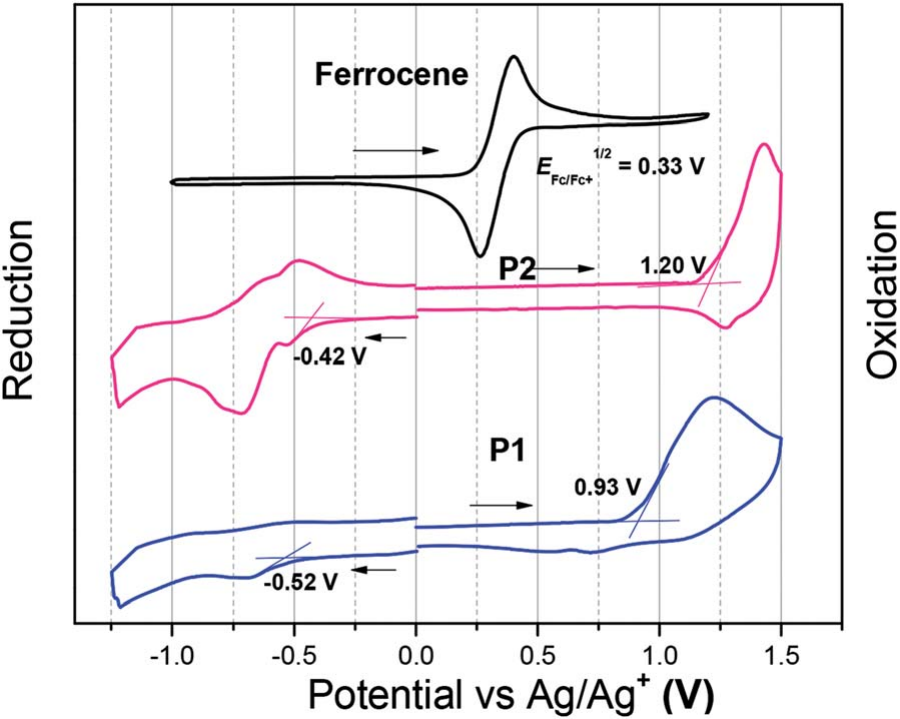
The reduction and oxidation curves of P1 and P2. The films were deposited from chloroform solutions.

The electrochemical properties of both polymers were determined using cyclic voltammetry (CV) from their drop-cast thin films (Fig. 2). The electron affinities (EAs) and ionization potentials (IPs) of the polymers were calculated from the onset of first reduction and oxidation potentials.^35^ The values of EA are 3.95 and 4.05 eV for P1 and P2, respectively, while the corresponding IP values are 5.40 and 5.67 eV. The electrochemical bandgaps of both polymers were thus deduced to be 1.45 and 1.62 eV for P1 and P2, respectively. The fluorinated polymer P2 thus has a larger electrochemical band gap, which is due to weaker donating effect of the T-FBT unit (lowered HOMO).

### 2.3 Quantum chemistry calculations

**Fig. 3:**
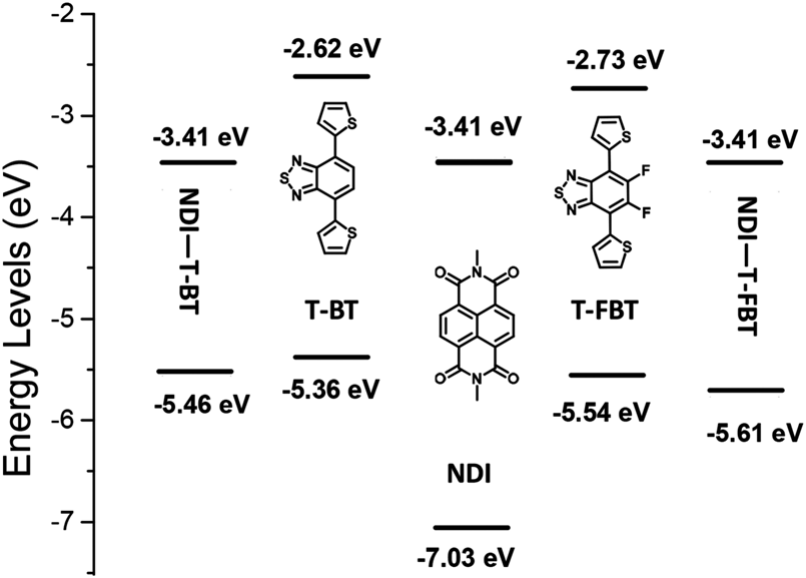
Energy level diagram of three monomers and the monomeric subunits of P1 and P2, the energy level values are obtained from DFT calculations (B3LYP, 6–31 g(d)).

The density functional theory (DFT) calculations were employed to better understand molecular energy levels of the monomers and polymers (Fig. 3).^36^ The alkyl chains were replaced by methyl substituents during calculation. The LUMO values are 3.41 eV for the monomeric subunits of P1 and P2, respectively, while the corresponding HOMO values are −5.46 and −5.61 eV. Interestingly, the weaker acceptors T-BT and T-FBT exhibit electron-donating nature in dimeric subunits of P1 and P2 (see Fig. S2†), respectively. Therefore, the LUMO energy levels of both polymers are determined by the strong acceptor NDI part, and their HOMO energy levels are contributed by T-BT and T-FBT moieties, respectively. The T-FBT has lower HOMO energy levels compared to T-BT. The energy level differences between NDI and T-FBT units are smaller than those of NDI and T-BT leading to a weaker ICT in NDI and T-FBT than that of NDI and T-BT. That is the reason why the monomeric subunits of P2 (2.20 eV) have a larger band gap than that of P1 (2.05 eV). The results from calculations are well consistent with the observations from optical absorption and CV confirming that the fluorinated polymer P2 has a significantly blue-shift and a larger bandgap compared to P1.

### 2.4 Self-organization

**Fig. 4:**
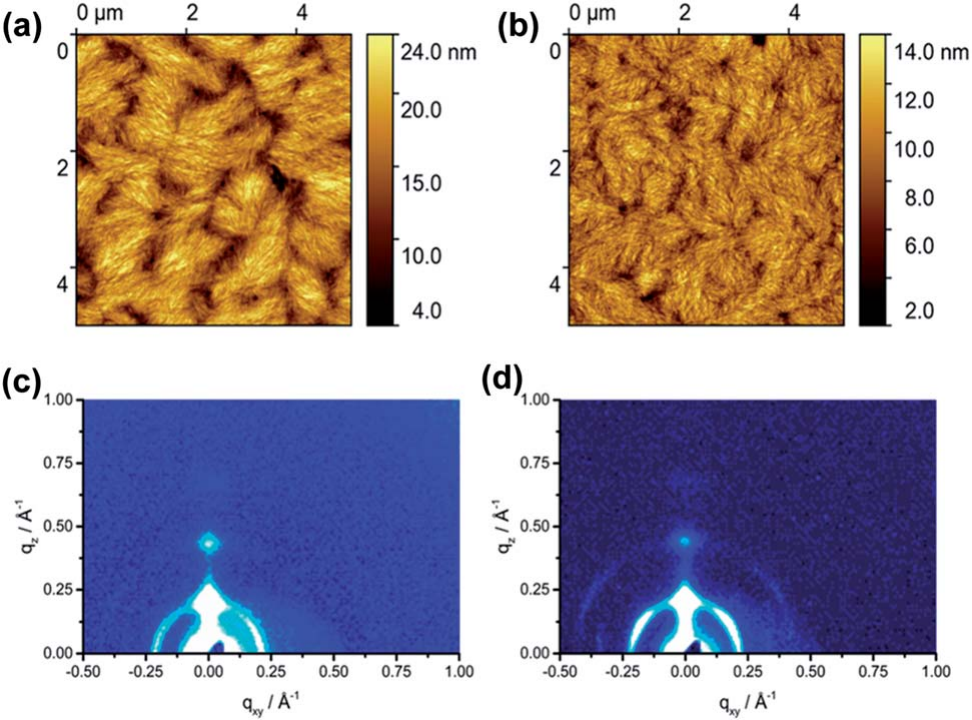
Tapping mode height AFM images of (a) P1 and (b) P2 thin films after annealing at 200 °C. Grazing-incidence wide-angle X-ray scattering (GIWAXS) patterns of (c) P1 and (d) P2 thin films after annealing at 200 °C.

The film microstructure of spin-coated P1 and P2 was investigated by tapping-mode atomic force microscopy (AFM) (Fig. 4) to find a correlation between supramolecular organization and film morphology. The 50 nm thick films were annealed at 120 °C to remove residual solvents. The film of P1 shows a fibrous microstructure with relatively large void areas and distinct grain boundaries between crystalline domains (Fig. S4a†). A surface area (SA) of 17.5 μm^2^ is determined for this film. This value describes the total area that the grains occupy in the film. Annealing at 200 °C slightly improves the microstructure leading to an interconnection between domains and increases SA to 20.2 μm^2^. The root-mean-square roughness (*R*_ms_) of the surface remains at the same level of 3.0 nm (Fig. 4a). Further annealing of P1 at 300 °C (Fig. S4b†) increases the roughness up to *R*_ms_ = 4.0 nm, whereby the size of domains stays almost unchanged (19.6 μm^2^) in comparison to annealing at 200 °C. Polymer P2 exhibited a less textured film topography with significantly reduced grain boundaries with respect to P1. The film of P2 annealed at 120 °C (Fig. S4c†) contains grains built from smaller fibers as expressed by a lower SA value of 15.1 μm^2^ and a lower roughness of *R*_ms_ = 1.5 nm compared to P1. Additional annealing at 200 °C (Fig. 4b) and 300 °C (Fig. S4d†) of P2 improves the interconnection between separated domains and slightly increases the surface area to 17.7 μm^2^, while the roughness persists on the same level. In summary of the AFM studies, only slight differences in surface morphology between P1 and P2 were found.

Grazing incidence wide-angle X-ray scattering (GIWAXS) of the spin-coated films was performed in order to investigate the effects of the F-containing units on the organization of the dual-acceptor polymers. After annealing at 120 °C, P1 reveals an out-of-plane 100 reflection at *q*_*z*_ = 0.24 Å^−1^ and *q*_*xy*_ = 0 Å^−1^ that corresponds to an interlayer distance of 2.61 nm of polymer chains indicating that P1 is preferentially arranged edge-on on the substrate (Fig. S3a†). An additional in-plane reflection at *q*_*z*_ = 0 Å ^−1^ and *q*_*xy*_ = 0.347 Å^−1^ is related to the monomeric unit length of the polymer backbone of 1.81 nm. This value is in agreement with model calculations. The increase in annealing temperature to 200 °C does not affect the position of the reflections, but slightly improves the long-range organization as confirmed by the appearance of higher order interlayer reflections (Fig. 4c). On the other hand, the higher temperature of 300 °C reduced the crystallinity again (Fig. S3b†). It has to be emphasized that P1 did not show any reflections corresponding to the π-stacking indicating poor intralayer molecular order. The GIWAXS pattern of P2 annealed at 120 °C exhibited similar structures as P1 (Fig. S3c†). An interlayer distance of 2.69 nm was found for P2, whereby two distinct surface orientations of the backbone with face- and edge-on are evident from the in-plane and out-of-plane positions of the corresponding reflections. Annealing at 200 °C and specially at 300 °C improved (Fig. S3d†) the organization of the polymer as confirmed by the appearance of higher order reflections. The in-plane reflection at *q*_*xy*_ = 0.347 Å ^−1^ and *q*_*z*_ = 0 Å ^−1^ is related to the *d*-spacing of 1.81 nm which corresponds to the theoretical calculated monomer length. Summarizing, GIWAXS results indicated that both polymers have a similar organization in thin films.

### 2.5 OFET properties

To find the relation between chemical polymer structure (BT and F-containing BT units) and charge carrier transport, P1 and P2 were employed as semiconducting thin films in organic field-effect transistors (OFET). The transistor characterization was performed after thermal annealing at 120 °C (to ensure evaporation of residual solvent), 200 °C and 300 °C. Highly doped silicon wafers were used as gate electrode, while the 300 nm thick thermally grown silicon oxide was surface modified by octadecyltrichlorosilane (OTS) to be exploited as the dielectric layer. Both polymers exhibit an ambipolar field-effect in a bottom-gate, top-contact configuration with gold electrodes. The best device parameters with the highest charge carrier mobility and lowest threshold voltages were observed for thin films thermally annealed at 200 °C (Table 1).

**Table 1:** Molecular weights, optical absorption, electrochemical properties and field-effect mobilities of P1 and P2

Polymer	*M*_n_ a (kg mol^−1^)	PDI^a^	*λ*_abs_ (nm) soln.^b^	*λ*_abs_ (nm) film^c^	*E* ^opt^_g_ d (eV)	IP^d^ (eV)	EA^d^ (eV)	*μ*_h, max_ (cm^2^ V^−1^ s^−1^)	*μ*_e, max_ (cm^2^ V^−1^ s^−1^)
P1	36.8	2.5	736	726	1.50	5.40	3.95	0.073	0.086
P2	44.0	2.8	697	679	1.62	5.67	4.05	2.4 × 10^−3^	0.258

a Determined by GPC in 1,2,4-trichlorobenzene using polystyrene standards at 135 °C.

b Dissolved in choloform (*c* = 10^−6^ M).

c Drop-cast from choloform solution (2 mg mL^−1^).

d IP and EA were estimated from the onsets of the first oxidation and reduction peak, while the potentials were determined using ferrocene (Fc) as standard by empirical formulas IP/EA = (*E*^onset^_Ox/Red_ − *E*_Fc/Fc^+^_^1/2^ + 4.8) eV wherein *E*_Fc/Fc^+^_^1/2^ = 0.33 V.

**Fig. 5:**
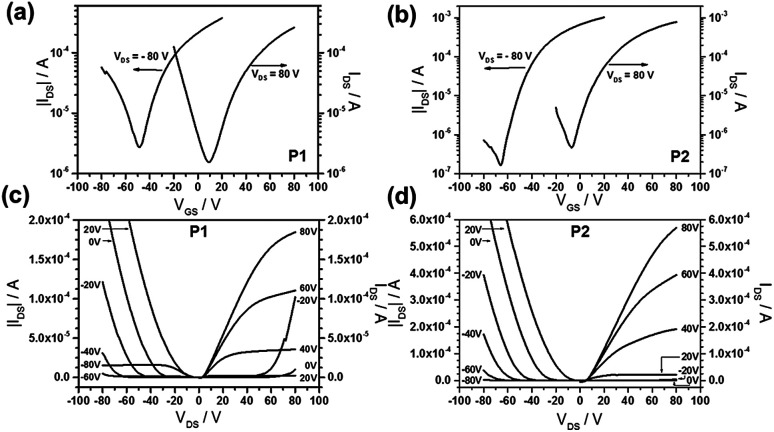
(a) and (b) Transfer, (c) and (d) output characteristics of P1 and P2 thin films after annealing at 200 °C.

Fig. 5 shows representative transfer and output characteristics for P1 (Fig. 5a and c) and P2 (Fig. 5b and d) after annealing at 200 °C. Polymer P1 exhibited a balanced ambipolar charge transport with mobilities of 0.073 cm^2^ V^−1^ s^−1^ and 0.086 cm^2^ V^−1^ s^−1^ for holes and electrons, respectively. These values are more balanced compared to previous work that hole and electron mobilities are 0.1 cm^2^ V^−1^ s^−1^ and 0.05 cm^2^ V^−1^ s^−1^, respectively, in the same device configuration,^29^ and less than 10^−3^ cm^2^ V^−1^ s^−1^ and 0.19 cm^2^ V^−1^ s^−1^, respectively, in bottom-gate bottom-contact device configuration.^19^ The ambipolar transport of P2 provided less balanced transport with an electron mobility of 0.258 cm^2^ V^−1^ s^−1^ and with a low hole transport of only 2.4 × 10^−3^ cm^2^ V^−1^ s^−1^. However, the same conjugated backbone of P2 was previously reported that the polymer only exhibits an electron molibility of 0.15 cm^2^ V^−1^ s^−1^ in bottom-gate bottom-contact device configuration.^19^ The decline in threshold voltage for electrons of P1 and P2 from 35 V to 6 V upon annealing at 200 °C is related to the enhancement of the polymer organization and film morphology described in the AFM and GIWAXS parts. Interestingly, after annealing at 200 °C, we have found for P1 a balanced ambipolar mobility with 10^−2^ cm^2^ V^−1^ s^−1^ for both types of carriers, while the electron mobility for P1 is one and for P2 two orders of magnitude higher in comparison to literature values for devices of the same structure.^19,29^ The electron mobility is higher in P2 in comparison to P1 what is attributed to the additional F atoms at the BT unit. The lower hole transport in P2 is assigned to the two strong acceptors lowering the HOMO of the polymer.

In top-gate bottom-contact device architectures, P1 and P2 backbones substituted by 2-decyl-tetradecyl side chains showed superior electron mobilities of 3.1 cm^2^ V^−1^ s^−1^.^19^ The reason for this improved electron transport is the polymer dielectric (PMMA) reducing the interfacial trapping in comparison to the inorganic SiO_2_ gate isolator.^37^ The difference in performance between P1 and P2 in our work can be explained as following. The metal/semiconductor interface is described as a Mott–Schottky barrier determined by the difference between the work function of the metal electrodes and the semiconductor HOMO or LUMO levels. When the work function is close to the HOMO or LUMO level of the semiconductor an ohmic contact is expected. Otherwise, the potential barrier significantly reduces the injection of the charges resulting in a change from a well-balanced to an imbalanced or even unipolar operation mode of the transistor. Optimization of the electrode work function towards the polymer energy levels is expected to favor a balanced electron and hole transport with mobilities above 1 cm^2^ V^−1^ s^−1^.^38^ As it was mentioned, the introduction of fluorine does not alter the LUMO level resulting in typical ohmic behavior. After improvement of the film microstructure by annealing, the threshold voltage for electron transport is reduced for both polymers down to 0 V. In literature, the lack of hole transport was mainly attributed to the mismatch of the HOMO level and Au work function.^19^ However, in our case the difference between work function of the Au electrodes (−5.1 eV) and IP of P1 (5.40 eV) is smaller than to the metal work function and IP of P2 (5.67 eV). In contrast, the difference between work function of the Au electrodes and EA of P1 (3.95 eV) is larger than to the metal work function and EA of P2 (4.05 eV) suggesting a more facile electron injection into P2. However, at the same time the hole injection would become more difficult into P2 in comparison to P1. This is the reason for the higher electron, but lower hole mobility of P2 than of P1.

## 3 Conclusions

In summary, we have presented two copolymers based on NDI and BT with a pair of 3-decyl-pentadecyl alkyl chains attached to the NDI unit. The introduction of fluorine atoms in the BT moiety plays a significant role on the optoelectronic and electrochemical properties. Compared to P1 without F atoms, the *λ*_max_ of P2 is blue-shifted, and the IP energy level of P2 is decreased more than their EA energy level. The introduction of 3-decyl-pentadecyl alkyl chains into NDI unit has an important influence on the device performance. Compared to the same conjugated backbone polymers, the FET device of P1 exhibits a well-balanced ambipolar charge carrier transport with hole and electron mobilities of 0.073 cm^2^ V^−1^ s^−1^ and 0.086 cm^2^ V^−1^ s^−1^, respectively. However, after introduction of F atoms into the BT unit, P2 shows a less balanced transport with an electron mobility of 0.258 cm^2^ V^−1^ s^−1^ and with a poor hole transport of 2.4 × 10^−3^ c cm^2^ V^−1^ s^−1^. The understanding the influence of fluorination and alkylation of NDI-BT copolymers is beneficial to further design dual-acceptor polymers towards well-balanced high-performance ambipolar transistors.

## Conflicts of interest

There are no conflicts to declare.

## Supplementary Material

RA-008-C8RA02915F-s001
