# Supplementary material for: Effect of fluorination of naphthalene diimide–benzothiadiazole copolymers on ambipolar behavior in field-effect transistors
Source: RSC Adv. 2018 May 3;8(30):16464–9. doi: 10.1039/c8ra02915f (PMC9080245; doi:10.1039/c8ra02915f)
Supplement: RA-008-C8RA02915F-s001 [file RA-008-C8RA02915F-s001.pdf]

Electronic Supplementary Material (ESI) for RSC Advances.
This journal is © The Royal Society of Chemistry 2018

Effect of Fluorination of Naphthalene Diimide-Benzothiadiazole Copolymers on Ambipolar Behavior in Field-Effect Transistors

Cunbin An,[a,b] Hanna Makowska,[a,c] Benlin Hu,[a] Ruomeng Duan,[a] Wojciech Pisula,[a,c] Tomasz Marszalek,[a,c] and Martin Baumgarten[a*]

[a] Max Planck Institute for Polymer Research, Ackermannweg 10, 55128 Mainz, Germany.

[b]present address: Institute of Chemistry, Chinese Academy of Sciences, Zhongguancun North First Street 2,100190 Beijing, PR China

[c] Department of Molecular Physics, Faculty of Chemistry, Lodz University of Technology, Zeromskiego 116, 90-924 Lodz, Poland.

Corresponding author: martin.baumgarten@mpip-mainz.mpg.de

Contents

Experimental section

(1) General Methods

(2) Substrate modification and OFET device fabrication

(3) Grazing Incidence Wide-Angle X-ray Scattering (GIWAXS)

(4) Synthetic details

Figure S1. TGA curves of **P1** and **P2**.

Figure S2 The DFT calculation for Dimer of **P1** and **P2** repeating units.

Figures S3 – S4. ^{1}H-NMR and ^{13}C-NMR spectra for **M1**.

Figures S5 – S6. ^{1}H-NMR spectra for **P1** and **P2**.

(1) General methods

^{1}H NMR and ^{13}C NMR spectra were recorded in deuterated solvents on a Bruker DPX 250. Matrix-Assisted Laser Desorption/ Ionization Time of Flight Mass Spectrometry (MALDI-TOF-MS) were

carried out in Max Planck Institute for Polymer Reserch, Mainz. Elemental analysis was carried out using a Foss Heraeus Vario EL in the Institute of Organic Chemistry at the Johannes Gutenberg-University, Mainz. UV–Vis-NIR absorption spectra were measured on a Perkin-Elmer Lambda 9 spectrophotometer at room temperature. Thermogravimetry analysis (TGA) were carried out on a Mettler 500 Thermogravimetry Analyzer. Cyclic Volatammetry (CV) was carried out on a computer-controlled GSTAT12 in a three-electrode cell in anhydrous acetonitrile solvents solution of Bu_4NPF_6 (0.1 M) with a scan rate of 50 mV/s at room temperature under argon. A Pt wire, a silver wire, and a glassy carbon electrode were used as the counter electrode, the reference electrode, and the working electrode, respectively. EA were estimated from the onsets of the first reduction peak, while the potentials were determined using ferrocene (Fc) as standard by empirical formulas EA = - (E_{Red}^{onset}-$E_{Fc/Fc+}^{1/2}$+4.8) eV. The molecular weights were determined by PSS-WinGPC (PSS) (pump: alliance GPC 2000) GPC equipped with an UV or RI detector running in 1,2,4-trichlorobenzene at 135 °C using a PLgel MIXED-B column (particle size: 10 mm, dimension: 0.8×30 cm) calibrated against polystyrene standards. Morphologies of polymer thin films were characterized by a Dimension Icon FS Atomic Force Microscopy (AFM) in tapping mode.

(2) Substrate modification and OFET device fabrication

The highly doped silicon wafers with a 300 nm thick thermally grown silicon oxide layer on top were used as substrates, which were ultrasonically cleaned with acetone and propan-2-ol and dry blown with nitrogen gas. Subsequently the Si/SiO_2 substrates were cleaned by immersion in piranha solution (a mixture of 3:7 (v/v) of 30% H_2O_2 and 98% H_2SO_4) for 30 minutes. The substrates were then rinsed few times with deionized water and methanol, dry blown with nitrogen gas and used immediately. The surface of SiO_2 was functionalized with a self-assembled monolayer (SAM) of octadecyltrichlorosilane (OTS) by immersing the substrates in an unstirred 0.5 % OTS solution in mixture of chloroform and hexane (1:4 (v/v)) for 30 minutes. After the modification, the substrates were rinsed by chloroform and dried with nitrogen gas.

The thin films of the polymers (**P1** and **P2**) were spin-coated on the OTS-modified Si/SiO_2 substrates using 8 mg/ml dichlorobenzene solutions at 1500 RMP for 60s. The solution and substrate temperature was 80 °C . In order to remove residual solvents all samples were annealed at 120 °C and then further annealed at 200 °C and 300 °C to improve the polymer organization. After deposition of

the semiconductor thin films, a bottom-gate top-contact configuration was applied for the OFET devices. Si and SiO_2 were used as the gate electrode and as the gate dielectric, respectively. The gold source and drain electrodes were deposited with a thickness of 100 nm by evaporation process using shadow masks. The OFET devices had a channel width (W) of 1 mm and a channel length (L) of 10, 15, 20, 25 and 30 μm. All electrical measurements (using Keithley 4200 SCS) were performed in a glovebox under nitrogen atmosphere.

(3) Grazing Incidence Wide-Angle X-ray Scattering (GIWAXS)

GIWAXS measurements of the thin films were performed at the Max Planck Institute for Polymer Research using a custom setup consisting of copper solid-anode X-ray tube (Bruker AXS Krystalloflex 760, operated at 35 kV and 30 mA), Osmic confocal MaxFlux optics and a three pin-hole collimation system. The X-ray beam size has 1.0 mm radius, and samples were irradiated just below the critical angle for total reflection with respect to the incoming X-ray beam (~0.1°). The scattering intensity was detected on a 2-D image plate (MAR-345) with a pixel size of 100 μm (2345 × 2345 pixels), and the detector was placed 300 mm from the sample center. Scattering data are expressed as a function of the scattering vector: $q=4\pi/\lambda \sin(\Theta)$, where Θ is a half the scattering angle and $\lambda=0.154$ Å is the wavelength of the incident radiation. Here q_{xy} (q_z) is a component of the scattering vector in plane (out-of-plane) to the sample surface. All X-ray scattering measurements were performed under vacuum (~1 mbar) to reduce air scattering and beam damage to the sample. All GIWAXS data processing and analysis was performed by using the software package Datasqueeze (http://www.datasqueezesoftware.com).

(4) Synthetic details

All chemicals and reagents were used as received from commercial sources without further purification unless stated otherwise. Chemical reactions were carried out under ambient atmosphere. Intermediates 2,6-dibromonaphthalene-1,4,5,8-tetracarboxylic acid (S1)[1] and 3-decylpentadecan-1-amine (S2)[2] were prepared according to the literature procedures.

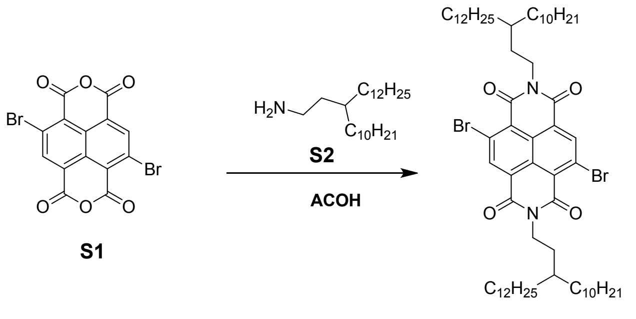

M1: 2,6-Dibromonaphthalene-1,4,5,8-tetracarboxylic acid dianhydride (**S1**, 1.0 g, 2.35 mmol)， 3-decylpentadecan -1-amine (**S2**, 1.73 g, 4.7mmol) and 40 mL of glacial acetic acid were added into two neck 100 mL flask under Argon. The reaction mixture was heated 120 ^{o}C with vigorous stirring. After two hours, the mixture was cooled to room temperature and poured into 100 mL 5% aqueous NaOH and extracted with dichloromethane. The combined organic phases were dried with $MgSO_4$ and filtered. The filtrate was concentrated and purified by column chromatography eluting with hexane dichloromethane (1:2) to give 1.05 g (yellow solid, 40%) of **M1**. ^{1}H NMR (250 MHz, CD_2Cl_2, ppm) δ 8.95 (s, 2H), 4.16 (m, 4H), 1.658 (m, 4H), 1.48 (br, 2H), 1.27 (br, 80H), 0.90-0.85 (m, 12H). ^{13}C NMR (62.5 MHz, CD_2Cl_2, ppm) δ 161.20, 161.14, 139.26, 128.44, 128.26, 126.01 124.72, 40.28, 36.51, 34.04, 32.49, 30.60, 30.27, 30.23, 29.94, 27.11, 23.26, 14.35 (Note: some peaks in ^{13}C NMR spectrum overlap). MALDI-TOF-MS (ESI+): m/z calc. 1125.3 found 1125.6.

Synthesis of P1

M1 (62.6 mg, 0.1 mmol), **M2** (112.5 mg, 0.1 mmol) and chlorobenzene (8 mL) were placed in a 50 mL two-neck flask. The mixture was purged with argon for 5 min, and then 5.5 mg of tris(dibenzylideneacetone)dipalladium(0) ($Pd_2(dba)_3$) and 7.3 mg of tri(*o*-tolyl)phosphine (P(*o*-tolyl)$_3$) were added. Then the mixture was heated up to 110 °C under argon. After 3 days, the polymer was end-capped with tributylphenylstannane and bromobenzene in sequence. After cooling to room temperature, the reaction mixture was added into methanol. The polymer was filtered and subjected to Soxhlet extraction with methanol, acetone, hexane, chloroform and chlorobenzene. The chlorobenzene fraction was collected and added 30 mL of sodium diethyldithiocarbamate aqueous solution (1g/ 100 ml) to remove residual Pd catalyst, the mixture was heated to 60 °C with vigorous stirring for 2 h. The mixture was separated and organic phase was washed with water for 3 times. The polymer was

collected from the chlorobenzene fraction and dried in vacuum to afford black solid (105 mg, 83 %). Molecular weight by GPC (135 °C): M_n = 36.8 KDa, PDI = 2.5. Anal. Calcd for C78H112N4O4S3: C, 74.00; H, 8.92; N, 4.43; S, 7.60. Found: C, 75.21; H, 8.64; N, 4.47; S, 7.94.

Synthesis of P2

P2 was prepared from **M1** and **M3** in similar procedure and workup to **P1**.

P2 (black solid, 104 mg, 80 %). Molecular weight by GPC (135 °C): M_n = 44.0 KDa, PDI = 2.8. Anal. Calcd for C78H110F2N4O4S3: C, 71.93; H, 8.61; N, 4.43; S, 7.21. Found: C, 71.96; H, 8.52; N, 4.30; S, 7.39.

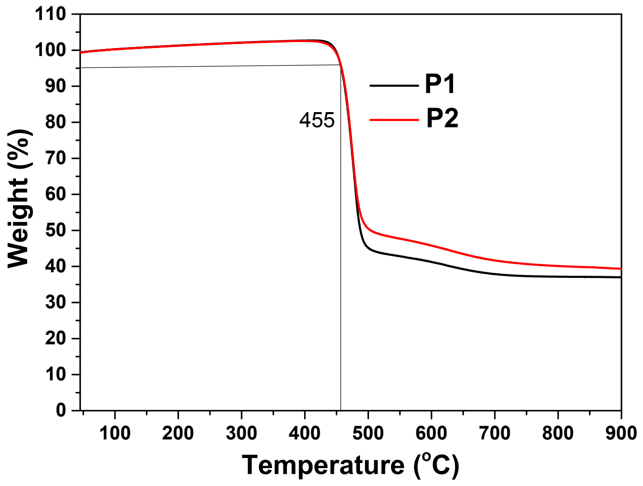

Figure S1. TGA curves for **P1** and **P2** measured under a nitrogen atmosphere at a heating rate of 10 ºC/ min.

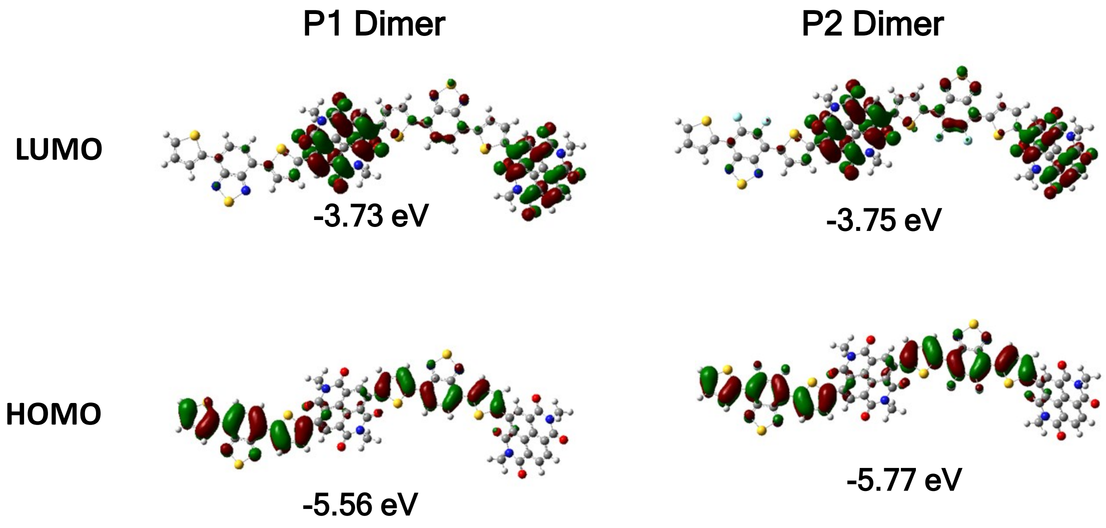

Figure S2. Energy-minimized structure (B3lyp/6-31G(d))[3] of a methyl substituted **P1** and **P2** dimer with the LUMO (top) and HOMO (bottom) energy distributions.

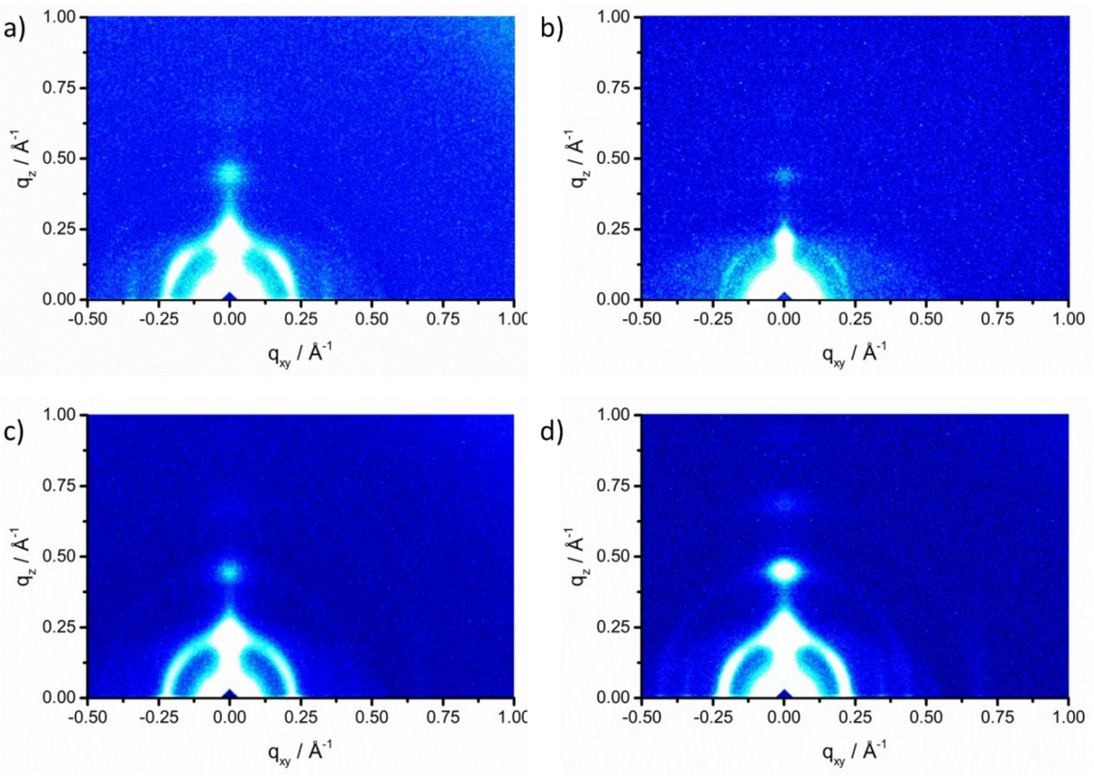

Figure S3. Grazing-incidence wide-angle X-ray scattering (GIWAXS) patterns of thin films of **P1** annealed at a) 120 °C and b) 300 °C, and **P2** annealed at c) 120 °C and d) 300 °C.

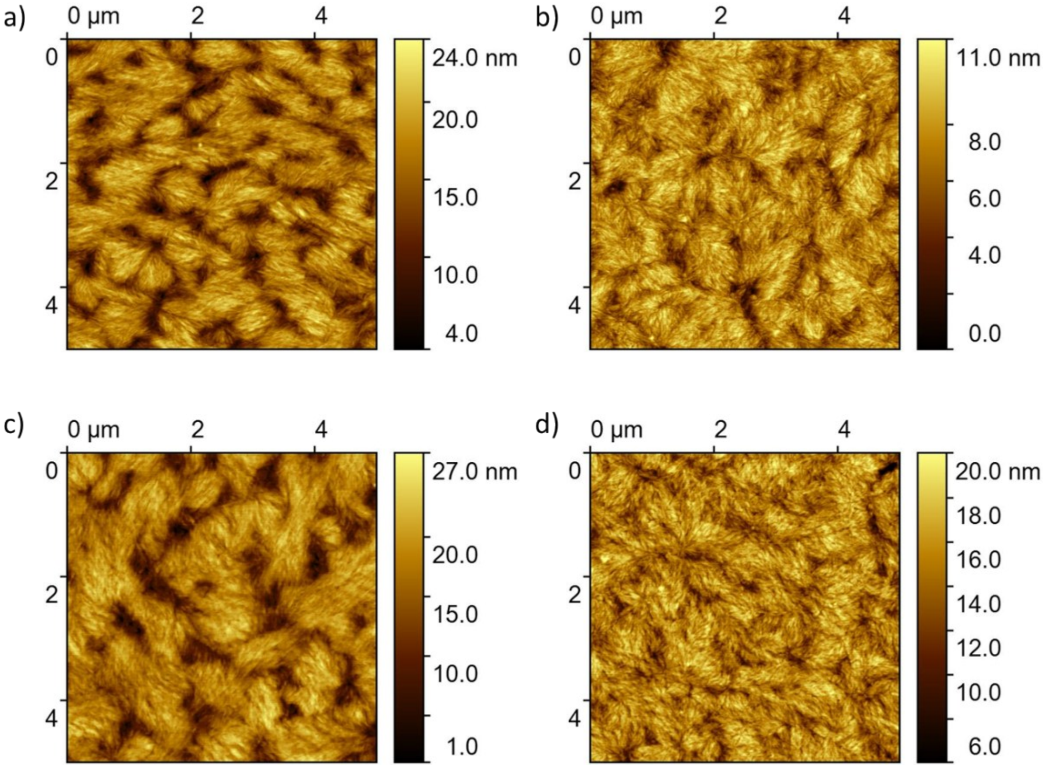

Figure S4. AFM height images of thin films of **P1** annealed at a) 120 °C and b) 300 °C, and **P2** annealed at c) 120 °C and d) 300 °C.

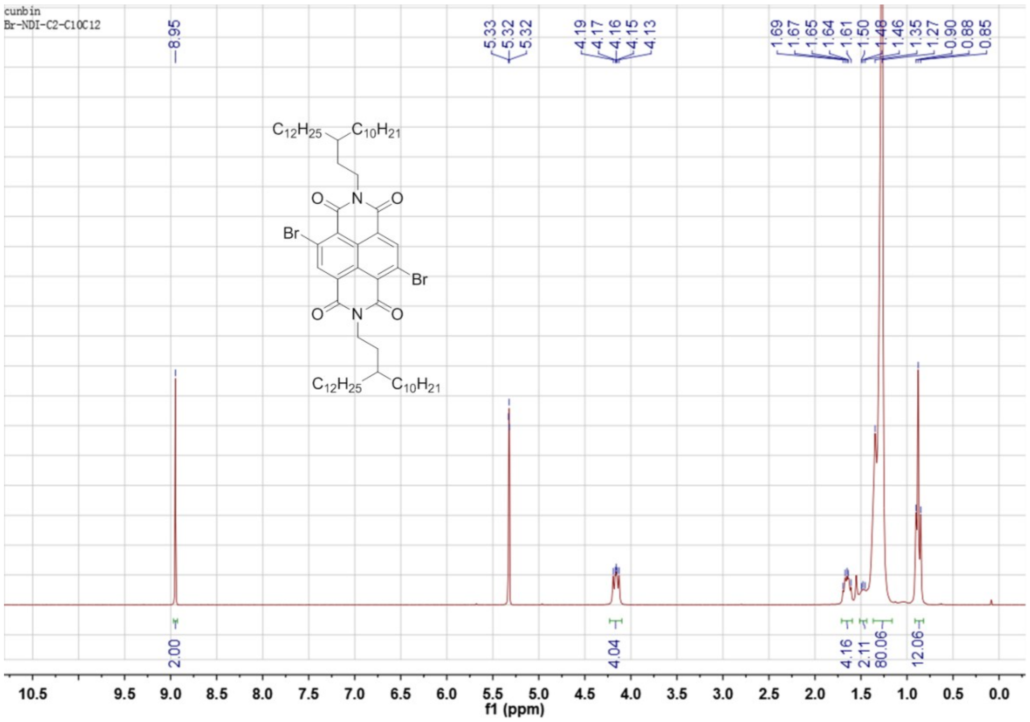

Figure S5. ^{1}H NMR spectrum of compound **M1**.

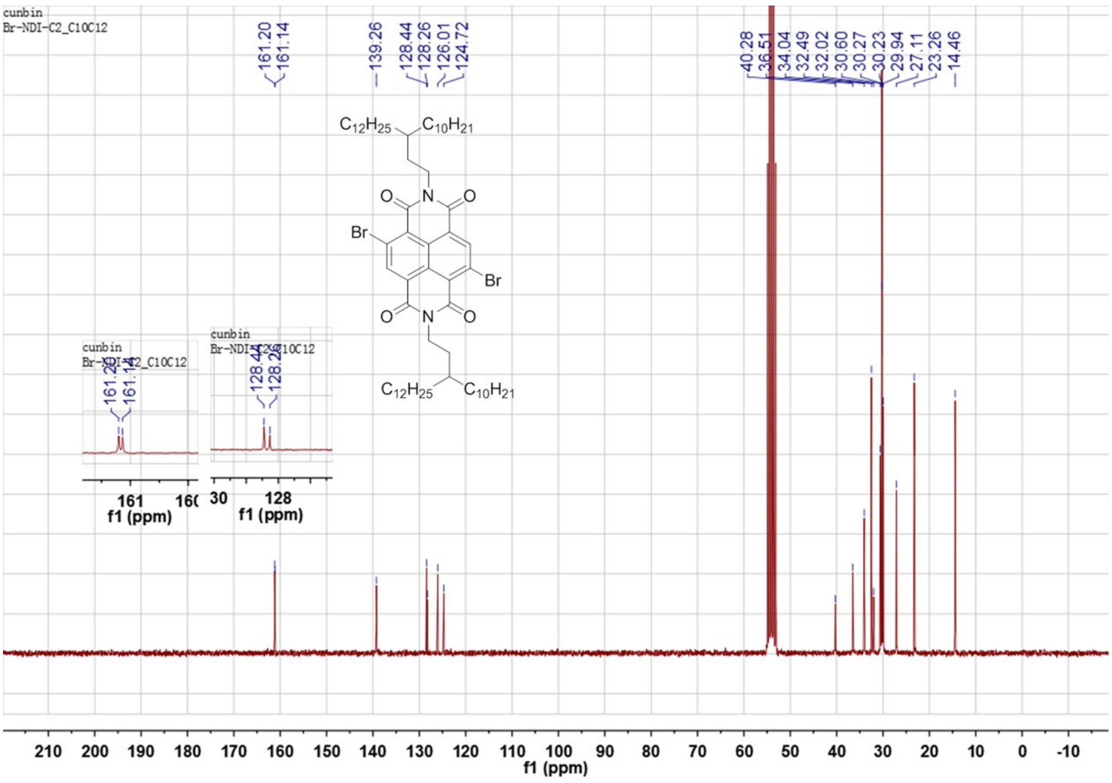

Figure S6. ^{13}C NMR spectrum of compound **M1**.

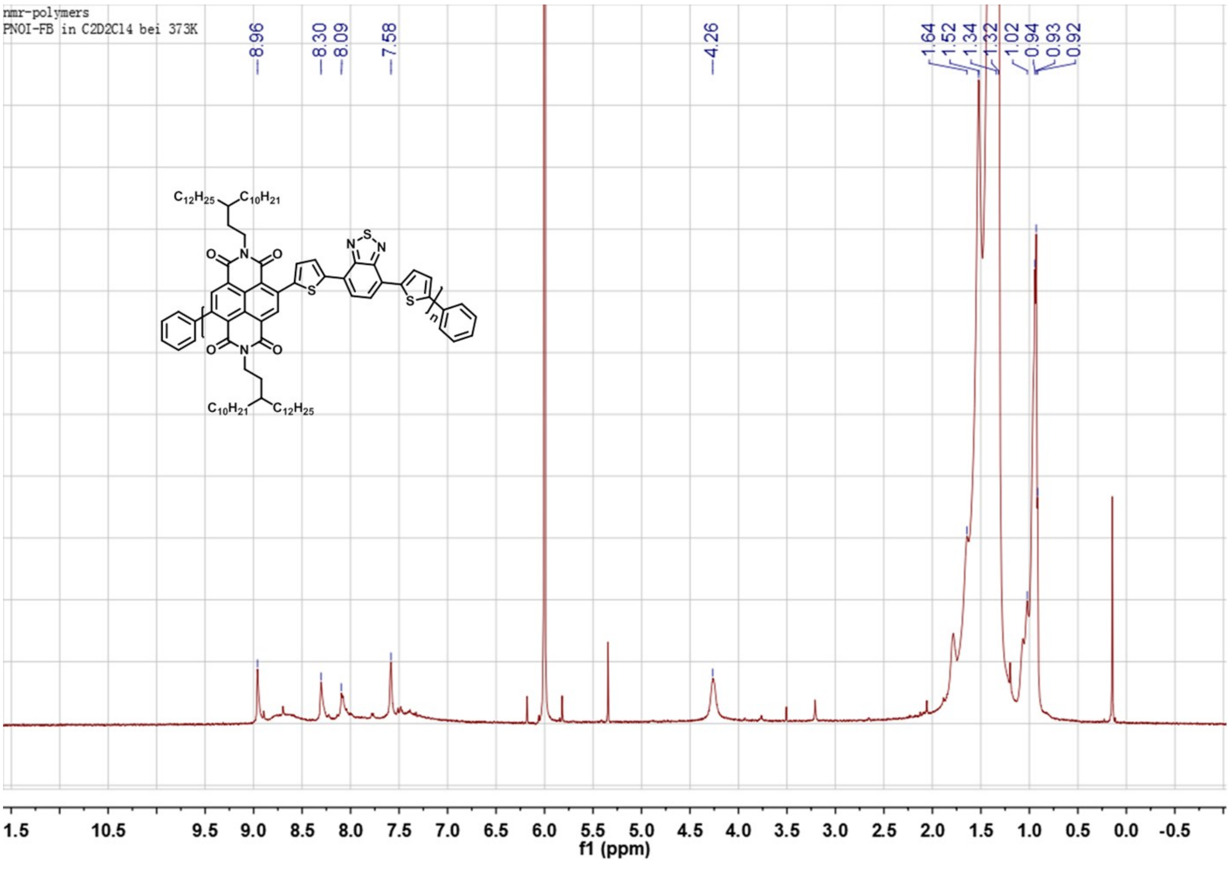

Figure S7. ^{1}H NMR spectrum of polymer **P1** in $C_2D_2Cl_4$ at 100 ^{o}C.

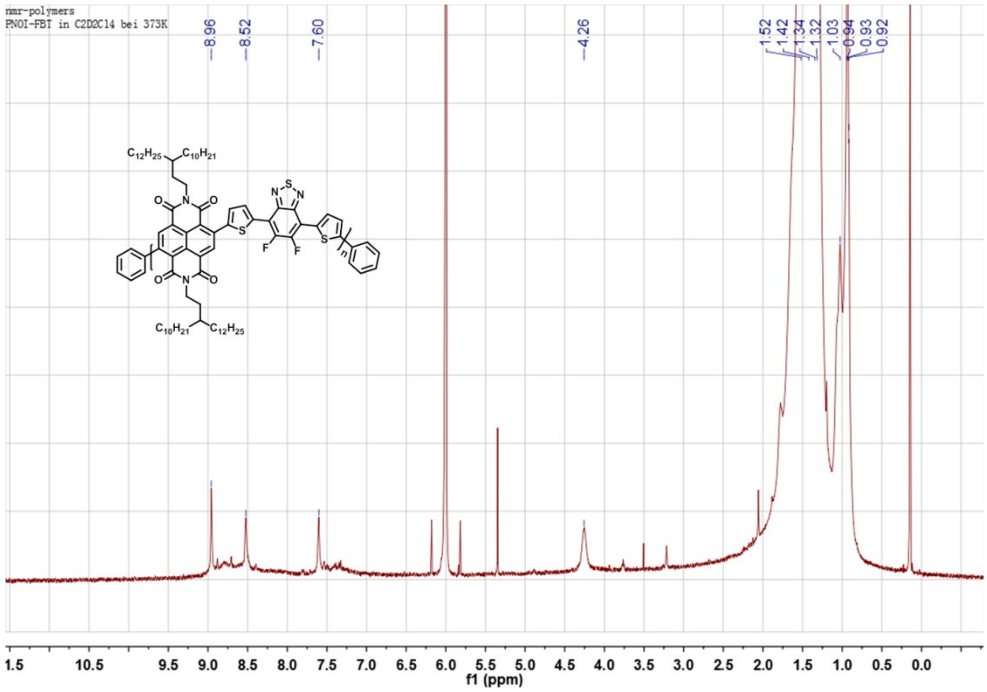

Figure S8. ^{1}H NMR spectrum of polymer **P2** in $C_2D_2Cl_4$ at 100 ^{o}C.

Reference

1. Guo, X.; Watson, M. D., Conjugated Polymers from Naphthalene Bisimide. *Org. Lett.* **2008,** *10* (23), 5333-5336.

2. Zhao, B.; Li, C.-Z.; Liu, S.-Q.; Richards, J. J.; Chueh, C.-C.; Ding, F.; Pozzo, L. D.; Li, X.; Jen, A. K. Y., A conductive liquid crystal via facile doping of an n-type benzodifurandione derivative. *J. Mater. Chem. A* **2015,** *3* (13), 6929-6934.

3. Frisch, M. J. et al. Gaussian 03, Revision B.04: Gaussian, Inc., Pittsburgh PA, 2003., Frisch, M. J. et al. Gaussian 03, Revision B.04: Gaussian, Inc., Pittsburgh PA, 2003.
